# Pseudorabies virus infection inhibits autophagy in permissive cells *in vitro*

**DOI:** 10.1038/srep39964

**Published:** 2017-01-06

**Authors:** Mingxia Sun, Linlin Hou, Yan-dong Tang, Yonggang Liu, Shujie Wang, Jingfei Wang, Nan Shen, Tongqing An, Zhijun Tian, Xuehui Cai

**Affiliations:** 1 State Key Laboratory of Veterinary Biotechnology, Harbin Veterinary Research Institute of Chinese Academy of Agricultural Sciences, Harbin, Heilongjiang Province 150001, P. R. China; 2 Department of Preventive Veterinary Medicine, College of Veterinary Medicine, Northeast Agricultural University, Harbin, Heilongjiang Province 150001, P. R. China

## Abstract

A large number of studies have demonstrated that autophagy is involved in the infection processes of different pathogens. Autophagy is now recognized as an essential component of innate and adaptive immunity. Several herpesviruses have developed various strategies to evade this antiviral mechanism. Pseudorabies virus (PRV) is a swine herpesvirus with a broad host range that causes devastating disease in infected pigs. In this study, we described the interaction between PRV and autophagy for the first time. PRV infection had a dual effect on the cell autophagy response; during the early period of infection, PRV virions induced autophagy without viral replication, and with viral protein expression, PRV reduced the basal level of autophagy in several permissive cells. We observed that inhibit the level of autophagy could increase the titer of infectious PRV. We also found that the conserved alphaherpesvirus US3 tegument protein may reduce the level of autophagy via activation of the AKT/mTOR pathways in PRV infected cells. These findings suggest that autophagy likely contributes to clearance of PRV, and that the virus has evolved strategies to antagonize this pathway.

Pseudorabies virus (PRV) is a swine herpesvirus in the *Alphaherpesvirinae* subfamily. PRV has a broad host range and can infect most mammals. However, pigs are the natural reservoir. PRV causes Aujeszky disease in infected adult pigs, which results in significant economic losses worldwide^1^.

Autophagy is an evolutionarily conserved catabolic process in eukaryotes during which lysosomes degrade cellular components, including long-lived proteins and organelles^2^,^3^,^4^. Autophagy is as an adaptive response to protect cells and organisms during periods of cellular stress. In addition, autophagy participates in cellular processes, such as homeostasis, clearance of intracellular pathogens, and immunity^5^,^6^. Emerging evidence suggests that autophagy plays an important role in viral pathogenesis^7^,^8^,^9^. Certain viruses can exploit autophagy for their benefit. Several RNA viruses, such as poliovirus and hepatitis C, require autophagic membranes to assemble their replication complexes in the cytoplasm^10^,^11^,^12^,^13^. Conversely, autophagy can be an antiviral defense mechanism. The term xenophagy describes the process through which the autophagy machinery protects eukaryotes from infection^14^. Activation of the autophagic pathway can effectively eliminate intracellular pathogens by fusing with lysosomes, which has been observed for bacteria, such as *Mycobacterium tuberculosis*^15^,^16^, and viruses, such as tobacco mosaic virus^17^ and herpes simplex virus^18^.

Studies with herpesviruses have contributed to our understanding of the significance of autophagy during virus infection. Herpesviruses contain several different proteins that interact with the autophagy machinery. The HSV-1 early neurovirulence protein ICP34.5 binds to the mammalian autophagy protein Beclin 1 to inhibit autophagy^19^. In addition, the HSV-1 late protein US11 inhibits autophagy without interacting with Beclin 1^20^. Orvedahl *et al*. demonstrated that a mutant HSV-1 lacking the Beclin 1-binding domain of ICP34.5 failed to inhibit autophagy in neurons and was less able to cause lethal encephalitis in mice. Similarly, Tallóczy quantified the number of virions per autophagosome in ICP34.5-null mutants and wild-type HSV-1 infected MEFs to demonstrate that ICP34.5 contributes to virulence by preventing the xenophagic destruction of virions^21^. Human cytomegalovirus (HCMV) has also been reported to inhibit the autophagy pathway in infected MRC-5 human fibroblasts^22^. However, a conflicting report demonstrated that autophagy was induced very early after infection with HCMV or HSV-1 and that de novo protein synthesis was not required for the response^23^. Kaposi’s sarcoma-associated herpesvirus (KSHV) has been shown to induce autophagy during lytic reactivation in a manner depending on the viral RTA transcription activator^24^. In addition, KSHV-encoded vBcl-2, vFLIP and K7 proteins inhibit autophagy at different formation stages, suggesting they have roles in the survival of transformed cells^24^,^25^,^26^,^27^. The functions of vBcl-2 in anti-autophagy and anti-apoptosis have important roles in the establishment of chronic infections and the reaction of the virus^26^. Thus, the role of autophagy in the pathogenesis of herpesviruses appears to be context specific.

PRV possesses several well-characterized methods to alter the host cell behavior to facilitate efficient viral propagation and to avoid the host immune response. For example, the viral protein US3 has been implicated in blocking apoptosis in infected cells in response to apoptotic stimuli^28^,^29^. However, PRV does not encode homologous genes to ICP34.5 or US11 of HSV-1, which antagonize the host’s autophagy response. To date, no clear interaction of PRV and the autophagy system has been observed, except for one report by Rasmussen that suggested PRV infection activates autophagy through DNA in nonpermissive cells^30^. Therefore, we aimed to investigate whether PRV can modulate the host autophagy response and how autophagy affects PRV infection. The studies reported here demonstrate that PRV infection has a dual effect on the cell autophagy response; during the early time of infection, PRV virions induce autophagy without the requirement of viral replication, and with viral protein expression, the autophagy response is inhibited. We observed that US3 could inhibit autophagy levels via activation of the AKT/mTOR pathways in PRV infected cells. Moreover, the autophagy response had a negative effect on viral infection.

## Results

### LC3 lipidation changed after infection with PRV in permissive cells

**Figure 1:**
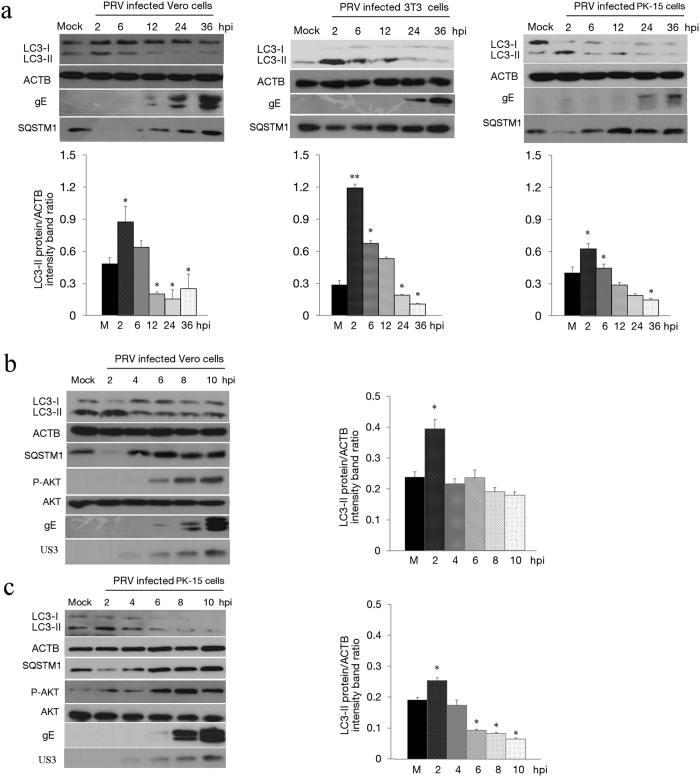
PRV infection modifies LC3 lipidation after infection in permissive cells. (**a**) Vero, 3T3 and PK cells were mock infected or infected with PRV at MOI of 0.1. At 2, 6, 12, 24 or 36 h post-infection (hpi), the cells were lysed, separated by reducing SDS-PAGE, and subjected to a Western blot using antibodies against LC3, ACTB (β -actin), SQSTM1 or PRV gE protein as indicated. Densitometry was performed for quantification. The ratio of LC3-II to ACTB from three independent experiments is expressed as the mean ± SD. One-way ANOVA test; *P < 0.05; **P < 0.01, as compared with the mock infection group. Vero cells (**b**) and PK cells (**c**) were mock infected or infected with PRV at MOI of 10. At 2, 4, 6, 8 or 10 hpi, the cells were lysed, separated by reducing SDS-PAGE, and subjected to a Western blot using antibodies against LC3, ACTB, SQSTM1, AKT, p-AKT (p-Ser473), PRV gE protein or US3 protein as indicated. Densitometry was performed for quantification. The ratio of LC3-II to ACTB from three independent experiments is expressed as the mean ± SD. One-way ANOVA test; *P < 0.05, as compared with the mock infection group. Gels were run under the same experimental conditions. For better clarity and concise presentation, cropped blots are shown. The raw uncropped images can be found in Supplementary Fig. S1.

To investigate whether PRV infection can alter the levels of autophagy, we investigated the status of the proteins LC3 and SQSTM1/p62 after the infection of Vero cells with PRV using a Western blotting analysis. Protein samples were prepared from harvested cells at increasing post-infection time intervals and were subjected to immunoblotting using an anti-LC3B antibody that recognized both forms of LC3. Meanwhile, antibodies against envelope protein E (gE) and tegument protein US3 of PRV were used to track the progression of virus infection. The expression levels of LC3-I and LC3-II were detected in PRV-infected Vero cells at 2, 6, 12, 24 and 36 h post-infection (hpi) (Fig. 1a). The autophagy level was represented as the ratio of LC3-II/ACTB band intensity. An increase in the intensity of LC3 II was detected by 2 hpi, and the extent of modification increased up to 6 hpi. The level of LC3-II was significantly reduced from 12 to 36 hpi in PRV-infected cells compared to the mock infected cells, which indicates that autophagy was inhibited during PRV infection in Vero cells. The PRV gE protein could be identified from 12 hpi in our experiment, which was consistent with the decrease in the level of LC3-II (Fig. 1a).

To further verify the autophagy activity in other PRV permissive cells, 3T3 cells (a fibroblast-type cell line) and PK-15 cells (a porcine cell) were used to quantify LC3-II levels during PRV infection. As shown in Fig. 1a, the levels of LC3-II significantly increased from 2 to 12 hpi and was then reduced until 36 hpi in 3T3 cells. The trends of LC3-II expression in PK-15 cells were similar to Vero cells, and the level of LC3-II increased up to 6 hpi and decreased thereafter.

According to the live cell fluorescence microscopy of PRV exocytosis and single-step growth kinetics, the first generation of PRV progeny virions is assembled at approximately 6 hpi^31^,^32^. To interpret the autophagy response in actively infected cells at the early stage of infection, infections with a multiplicity of infection (MOI) of 10 were performed in Vero cells and PK-15 cells from 0 to 10 hpi. Our results showed that LC3-II expression increased at 2 hpi and then decreased after 4 hpi. PRV US3 protein was detected after 4 hpi by Western blotting and was consistent with the reduction of LC3-II; gE was detected from about 6 or 8 hpi after LC3-II decreased (Fig. 1b,c). The level of autophagy marker proteins for LC3-II decreased progressively with increasing infection time. These findings indicate that autophagy was activated in the early stages of PRV infection and was then inhibited during virus replication.

### Further analysis of autophagy in PRV-infected cells

**Figure 2:**
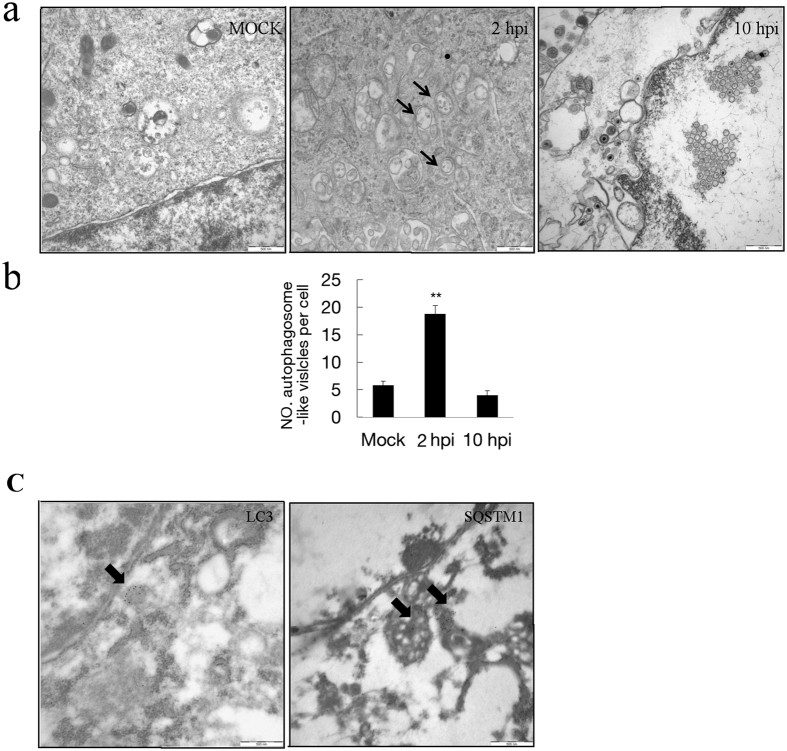
The transmission electron microscopy analysis of virus infected cells. (**a**) PK-15 cells were mock infected or infected with PRV at an MOI of 10 for 2 and 10 h. The cells were then fixed and processed for EM analysis. The vesicles with the characteristics of autophagosomes are indicated by black arrows in the relevant parts. Scale bars, 500 nm. (**b**) The quantification of the number of autophagosome-like vesicles per cell in mock and PRV infected cells is shown. Error bars indicate the mean ± SD for 30 cells per experimental condition of three independent experiments. One-way ANOVA test; **P < 0.01, as compared with the mock infection group. (**c**) The cells were processed for IEM analysis. SQSTM1 and LC3 were visualized with specific antibodies and detected with a secondary antibody conjugated to 18-nm colloidal gold particles. The immunogold labeling localized to the infection-associated membranes, as indicated by the black arrows.

Additional evidence for the induction of autophagy by PRV was obtained by examining PK-15 cells infected with PRV using transmission electron microscopy (TEM)^33^. We examined the formation of autophagosome-like vesicles in PRV-infected PK-15 cells with an MOI of 10 from 2 hpi until 10 hpi and performed a quantitative analysis. Increased double- or single-membrane vesicles were observed in PRV-infected PK-15 cells around the perinuclear region at 2 hpi, while similar vesicles were rarely seen in the uninfected cells and PRV-infected cells at 10 hpi (Fig. 2a). Further quantitative analyses demonstrated that there was a significant increase in the number of autophagosome-like vesicles in the cytoplasm of PRV-infected cells at 2 hpi and a decrease in the PRV-infected cells at 10 hpi (Fig. 2b). The number of autophagosome-like vesicles was consistent with the change of the lipidation of protein LC3, which suggested that PRV infection first induced and subsequently blocked autophagy.

To confirm that the double-membrane vesicles indeed represented autophagosomes, immunoelectron microscopy was performed with autophagosome markers LC3 and SQSTM1. As shown in Fig. 2c, the expression of LC3 was observed, and immunogold labeling was localized around the electron-dense membranes. For the expression of autophagy protein SQSTM1, the immunogold labeling was localized in electron-dense areas. The negative controls, which were incubated with normal rabbit IgG without the primary antibody, showed no positive signals.

**Figure 3:**
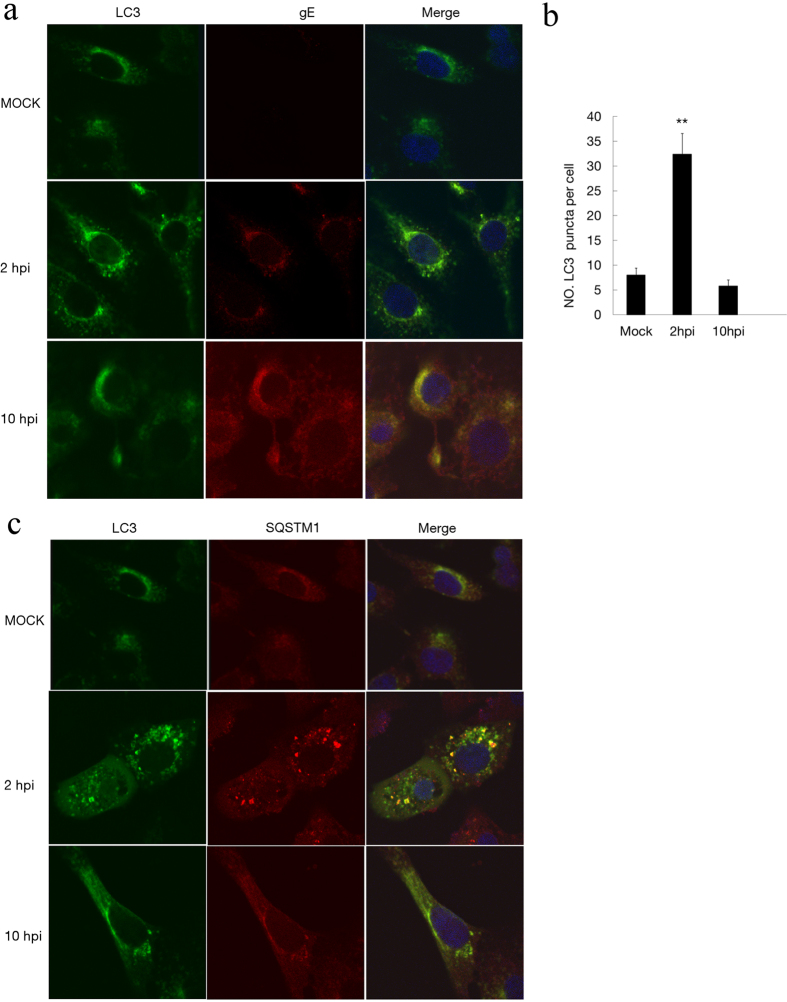
The formation of autophagosome-like vesicles in PRV-infected cells. (**a**) Vero cells were mock infected or infected with PRV at an MOI of 10 for different times. Then, the cells were fixed and incubated with anti-LC3 antibody and anti-gE antibody in subsequence, followed by the corresponding secondary antibodies conjugated to FITC and TRITC as described in the Methods. The cell nuclei were counterstained with DAPI. Immunoconfocal microscopy analyses were performed. (**b**) The number of cells showing LC3 dots was counted, and at least 100 cells were included for each group. Error bars indicate the mean ± SD for 100 cells per experimental condition of three independent experiments. One-way ANOVA test; **P < 0.01, as compared with the mock infected group. (**c**) LC3 colocalization with SQSTM1 was assessed by immunoconfocal microscopy analysis. Vero cells were processed as described in the Methods section. The cell nuclei were counterstained with DAPI.

The localization of LC3 in infected Vero cells was visible using the anti-LC3 antibody for immunoconfocal microscopy analysis. The LC3 can be redistributed from a diffuse cytoplasmic localization to a distinctive puncta cytoplasmic pattern during autophagy, which reveals the recruitment of LC3 to autophagic vesicles^34^. Vero cells were infected with PRV at an MOI of 10, and at 2 and 10 hpi, the cells were fixed and subjected to immunoconfocal microscopy analyses. The result showed that LC3 proteins were distributed as foci in most of the PRV-infected Vero cells at 2 hpi. LC3 puncta could co-localize with gE protein in PRV-infected cells. These puncta were reduced at 10 hpi along with gE protein expression (Fig. 3a). The number of LC3 puncta per cell was quantified (Fig. 3b). To confirm that these puncta were autophagosomes, the secondary marker SQSTM1 was used and analyzed by immunoconfocal microscopy. As shown in Fig. 3c, SQSTM1 was co-localized with LC3 puncta in PRV-infected cells. Taken together, the microscopy studies provided strong evidence that autophagy occurred in response to PRV in the early stages of infection.

### PRV infection enhances autophagy flux

Autophagosomes are transient vesicles that deliver their cargo by fusing with lysosomes for degradation within minutes. The increased lipidated LC3-II and the autophagosomes observed may result from an autophagosome formation increase or a decreased fusion with lysosomes for degradation. Studies have demonstrated autophagic degradation functions play an important role in the virulence of HSV^21^. To determine the autophagic degradation during PRV infection, SQSTM1/p62, a marker for the autophagy-mediated protein degradation pathway, was analyzed using immunoblotting^33^,^35^,^36^. As shown in Fig. 1, PRV infection caused the degradation of SQSTM1 during the period of increased autophagic levels, which suggests that the degradation function of autophagy was not affected by PRV.

**Figure 4:**
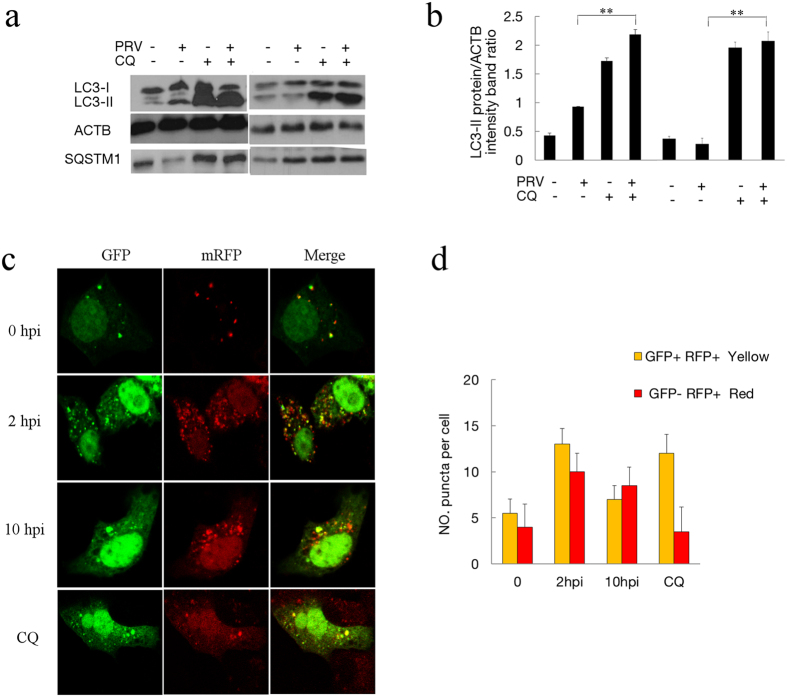
PRV infection enhances autophagy flux. (**a**) The modification of LC3 in mock-infected or PRV-infected Vero cells treated with CQ. Vero cells were mock infected or infected with PRV at an MOI of 10. CQ was pretreated for 4 h. At 2 and 8 hpi, cell samples were harvested and lysed, and the cell extracts were analyzed by immunoblotting using anti-LC3 and anti-SQSTM1 antibodies. (**b**) The ratio of the intensity of LC3-II to ACTB was calculated to represent the autophagic level. The data represent the mean ± SD of three independent experiments. One-way ANOVA test; **P < 0.01. Gels were run under the same experimental conditions. For better clarity and concise presentation, cropped blots are shown. The raw uncropped images can be found in Supplementary Fig. S2. (**c**) The colocalization analysis of mock-infected Vero cells and cells infected with PRV at an MOI of 10 for different times after transfection with GFP-mRFP-LC3. The cells were fixed and analyzed by confocal microscopy. (**d**) The graph shows the quantification of autophagosomes and autolysosomes by calculating the average number of dots in 20 cells. Autolysosomes were quantified by counting the number of RFP puncta per cell, and autophagosomes were quantified by counting the number of Yellow puncta per cell. The results are the means of three independent experiments. Error bars indicate the mean ± SD for 20 cells per experimental condition of three independent experiments.

In order to confirm the complete autophagy pathway and degradation, chloroquine (CQ) was used to analyze the turnover of autophagosomes. The upregulation of LC3-II in the presence of lysosomal protease inhibitors represents increased autophagic flux. The PRV infected and mock infected cells were pre-treated with CQ for 4 h. The expressions of LC3-II and SQSTM1 were assessed and quantified at 2 and 8 hpi. We first performed an experiment at 2 hpi when cells were in the autophagy induction stage. In uninfected cells, LC3-II accumulated after CQ treatment, indicating that autophagosome turnover and the degradation of LC3-II by lysosomal proteolysis was inhibited. In the infected cells, an accumulation of LC3-II was also observed in response to CQ treatment. In CQ-treated cells, the level of SQSTM1 was notably increased regardless of infection, which confirmed the effect of lysosomal protease inhibition of CQ. We then tested the autophagy level at 8 hpi when cells were in the autophagy inhibition stage. LC3 increased in both mock and PRV-infected cells under CQ treatment, which was the same as at 2 hpi. These results demonstrated that autophagy flux was present in the PRV-infected cells (Fig. 4a).

We also investigated autophagic flux in PRV-infected cells using the mRFP-GFP tandem fluorescent-tagged LC3 plasmid^37^. The GFP moiety of this tandem is sensitive to lysosomal proteolysis and quenching in acidic pH, but the RFP is not. Therefore, this probe makes it possible to differentiate between autophagosomes (GFP-positive and RFP-positive, which show yellow puncta) and autolysosomes (GFP-negative and RFP-positive, which show red puncta). The numbers of red puncta and yellow puncta per cell in mock-infected or PRV-infected cells and CQ treated cells at different time points post-infection were statistically analyzed. In uninfected cells, there were a few yellow autophagosomes and red autolysosomes after transfection with mRFP-GFP-LC3. The number of red puncta per cell, corresponding to autolysosomes, was clearly larger in PRV-infected cells at 2 and 10 hpi compared to mock-infected cells, indicating that autophagosomes fused with lysosomes. In PRV-infected cells at 10 hpi, the number of autophagosomes and autolysosomes was reduced with more autolysosomes, suggesting that the autophagy response decreased without inhibition of autophagy flux in PRV-infected cells at 10 hpi. In CQ-treated cells, which have suppressed autophagosome and lysosome fusion, there were only a few red autolysosomes, but a large number of yellow autophagosomes were detectable (Fig. 4c,d). The results showed that PRV infection of mRFP-GFP-LC3 transfected cells resulted in a shift from yellow to partially red fluorescence from 0 to 10 hpi. These data suggested that PRV infection enhanced the autophagic flux along with the entire process of infection.

### Replication of the virus is required to inhibit autophagy but not to induce autophagy

**Figure 5:**
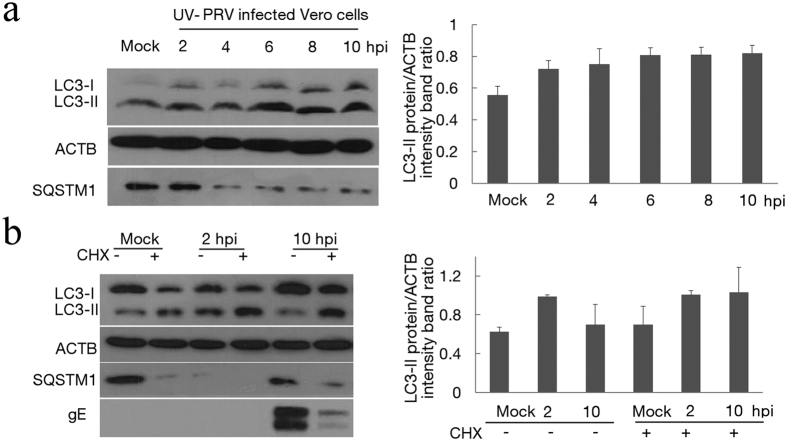
The relationship between autophagy and viral replication. (**a**) The modification of LC3 in UV-PRV-infected Vero cells. Vero cells were mock infected or infected with UV-PRV at an MOI of 10. At 2, 4, 6, 8 and 10 hpi, the cell samples were harvested and lysed, and the cell extracts were analyzed by immunoblotting using the anti-LC3 antibody. The ratio of intensity of LC3-II to ACTB was calculated to represent the autophagic level. The data represent the mean ± SD of three independent experiments. (**b**) The modification of LC3 in PRV-infected Vero cells in the presence of cycloheximide (CHX). At 2 and 10 hpi, the cell samples were harvested and lysed, and the cell extracts were analyzed by immunoblotting using anti-LC3 and anti-gE antibodies. The ratio of the intensity of LC3-II to ACTB was calculated to represent the autophagic level. The data represent the mean ± SD of three independent experiments. Gels were run under the same experimental conditions. For better clarity and concise presentation, cropped blots are shown. The raw uncropped images can be found in Supplementary Fig. S3.

To better characterize autophagy in PRV-infected cells, we next quantified the status of LC3 after infection of Vero cells with PRV from 0 hpi. The level of LC3-II increased from 30 min post-infection to before the expression of viral protein. Thus, the requirement for viral gene expression was investigated by infecting cells with a UV- inactivated PRV sample. The inactivated PRV was confirmed by titration and immunofluorescence assay (Supplemental Fig. S7). As shown in Fig. 5a, cells infected with UV-inactivated PRV had a higher level of LC3-II compared with the mock-infected cells with the effect detectable at 2 hpi and persisting until 10 hpi. This result suggested that viral gene expression was not required to induce autophagy. Compared to the low activity autophagy response in PRV-infected Vero cells at 4 hpi, there was no decrease in autophagy after 4 hpi in UV-inactivated PRV infected cells, which also suggested that PRV replication was required to inhibit autophagy.

The requirement of protein synthesis to induce autophagy was tested by carrying out infection in the continuous presence of cycloheximide (CHX) to inhibit protein translation. The results presented in Fig. 5b demonstrated that CHX treatment during PRV infection decreased the expression of gE protein and increased the autophagy level in PRV-infected cells at 10 hpi. Importantly, our results in Fig. 1 show that the decreased tendency of the LC3-II level was consistent with the expression of gE protein. As a whole, the data demonstrate that the virus particles could induce the autophagy response even in the absence of de novo protein synthesis, while replication of the virus was necessary to inhibit autophagy.

### Autophagy is involved in PRV infection

**Figure 6:**
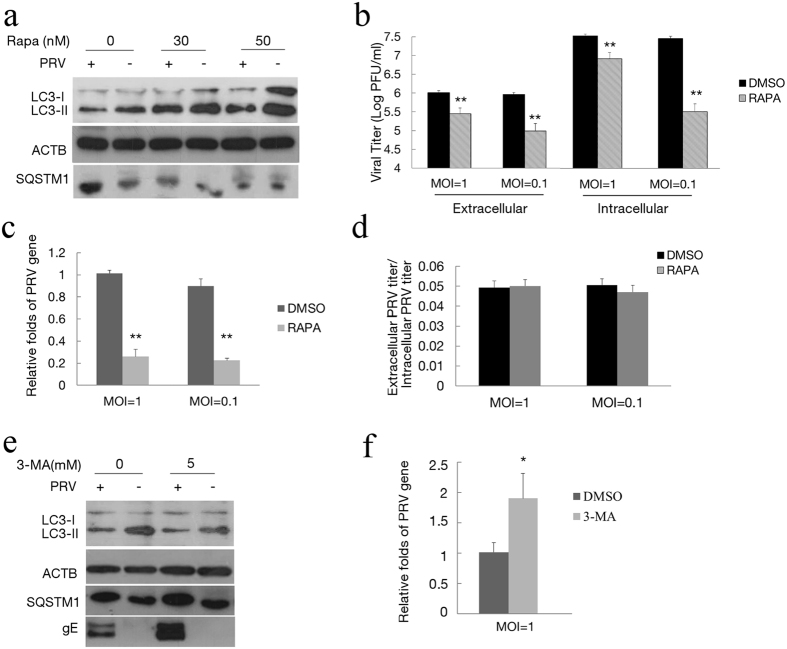
Autophagy is involved in PRV infection. (**a**) Vero cells were infected with PRV and then treated with rapamycin at 6 hpi, and the level of autophagy caused by rapamycin in the PRV-infected cells was tested by immunoblotting at 24 hpi. The effects of rapamycin on the replication of PRV were assayed by PFU assay (**b**) and Q-PCR (**c**) as described in the Methods section. (**d**) The ratio of the virus titers in supernatants and cell lysates was determined according the results of (**b**). (**e**) Vero cells were pretreated with 3-MA for 4 h and then infected with PRV. The level of autophagy and gE expression were assessed by immunoblotting at 24 hpi. (**f**) The replication of PRV was assayed by Q-PCR as described before. Gels were run under the same experimental conditions. For better clarity and concise presentation, cropped blots are shown. The raw uncropped images can be found in Supplementary Fig. S4.

Given that PRV infection decreases cellular autophagy activity along viral replication, we then determined whether autophagy involves in PRV replication. To accomplish this measurement, we exposed the cells to rapamycin to increase the autophagy level. Rapamycin was added to PRV-infected cells for additional 24 h incubation at 6 hpi, and then the level of autophagy was tested. As shown in Fig. 6a, treatment of rapamycin could partially increase the autophagy level in PRV-infected cells. The virus yield was analyzed at 24 hpi, and culture supernatant and intracellular viral titers from the infected cells were determined by a plaque forming unit (PFU) assay. According to the results of PFU, the virus yield decreased both in extracellular and intracellular fractions of infected cells (Fig. 6b). Notably, the effect of rapamycin treatment on the extracellular viral titer was similar to the intracellular titer with no significant changes in the ratios between them (P > 0.05) (Fig. 6d). The level of the PRV gB gene in the total infected Vero cells from the culture supernatant and intracellular fraction was evaluated by Q-PCR. As shown in Fig. 6c, the expression of the PRV gB gene in rapamycin treated cells decreased. These dates suggested that autophagy reduced the infection of PRV and did not influence viral release.

We then exposed the cells to 5 mM of 3-MA to decrease autophagy levels and assess the role of autophagy in PRV replication. The effect of 3-MA on autophagy and PRV replication were detected by Western blot analysis. As shown in Fig. 6e, treatment of 3-MA decreased the autophagy level and increased the expression of the gE protein. The expression of the PRV gB gene in 3-MA treated cells was increased 2-fold, as evaluated by Q-PCR (Fig. 6f). These dates suggested that decreased autophagy could prompt the replication of PRV.

**Figure 7:**
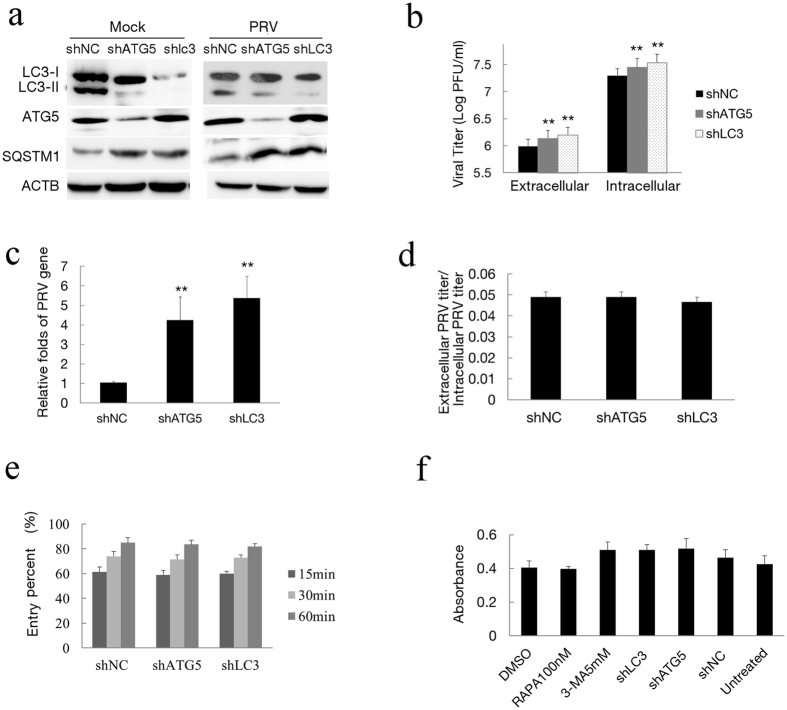
Inhibition of autophagy with specific shRNA targeting endogenous autophagy genes enhances PRV replication. (**a**) Vero cells were transfected with shRNAs to knockdown the autophagy proteins ATG5 and LC3B for 24 h. The cells were then mock infected or infected with PRV for 24 h. Western blot analysis was performed to monitor the expression of ATG5 and LC3B protein and autophagic levels. (**b**) The viral titer determined by PFU assay and the level of the virus gene gB from the treated cells (**c**) was evaluated by Q-PCR as described in the Methods section. (**d**) The ratio of the virus titers in supernatants and cell lysates were determined according the results of (**b**,**e**) To investigate the effects of ATG5 and LC3B knockdown on virus entry, cells were transfected with shRNA for 24 h and then infected with 100 PFU of PRV. Plaques were counted as described in the Methods section. (**f**) Cytotoxicity was determined by measuring lactate dehydrogenase (LDH) release from cells with a cytotoxicity assay kit according to the manufacturer’s instructions, and the absorption at 490 nm was measured. Vero cells were transfected with a shRNA vector for 48 h, and a cytotoxicity assay was also performed. One-way ANOVA; *P < 0.05 and **P < 0.01, as compared with the control group. Gels were run under the same experimental conditions. For better clarity and concise presentation, cropped blots are shown. The raw uncropped images can be found in Supplementary Fig. S5.

To confirm that autophagy inhibits PRV infection, we next examined PRV replication using target-specific RNA interference to reduce the endogenous autophagy proteins ATG5 or LC3B. Vero cells were transfected with ATG5 or LC3B shRNA plasmids designed to specifically silence the expression of these two genes. As shown in Fig. 7a, the level of endogenous ATG5 protein was reduced significantly in transfected Vero cells compared with the cells transfected with negative control and LC3B shRNAs. The reduced LC3-II expression and SQSTM1 degradation indicated a lower autophagy condition in the cells transfected with ATG5 and LC3B shRNAs (Fig. 7a). To ensure efficient infection to transfected cells, an MOI of 2 was used to improve the infection ratio in shRNA experiments. We observed an increased viral titer in extracellular and intracellular fractions of the cultures after autophagy protein silencing (Fig. 7b). Meanwhile, the ratios of the viral titers in the supernatants and cell lysates were similar between ATG5 and LC3 knockdown cells and the control cells (P > 0.05), as determined by the PFU assay, indicating that ATG5 and LC3 knockdown did not affect the viral release (Fig. 7d). The knockdown of ATG5 and LC3 resulted in an increase in virus genes according to Q-PCR analysis (Fig. 7c). To determine the effects of autophagy on virus entry, we analyzed the entry presences of shRNA transfected cells at different times of penetration according to methods that were previously described^38^,^39^. The results in Fig. 7e show that greater than 80% of viruses could enter cells and form plaques when cells were incubated for 1 h. There were also no differences in the entry percentage among three shRNA-transfected cells. Collectively, these results revealed that knockdown of the endogenous ATG5 and LC3B genes increased the infection of PRV.

In addition, we measured the viability of rapamycin or 3-MA treated cells and ATG5 or LC3B depleted cells using the CytoTox-One homogenous membrane integrity kit after transfection according to the manufacturer’s instructions. Our results demonstrated that the viability of Vero cells was not significantly affected after rapamycin or 3-MA treatment. The viability of ATG5 and LC3B depleted cells was close to that of untreated cells (Fig. 7f). Collectively, these results revealed that autophagy had a negative effect on PRV infection.

### PRV tegument protein US3 inhibits the autophagy response by activating PI3-K/AKT pathways

**Figure 8:**
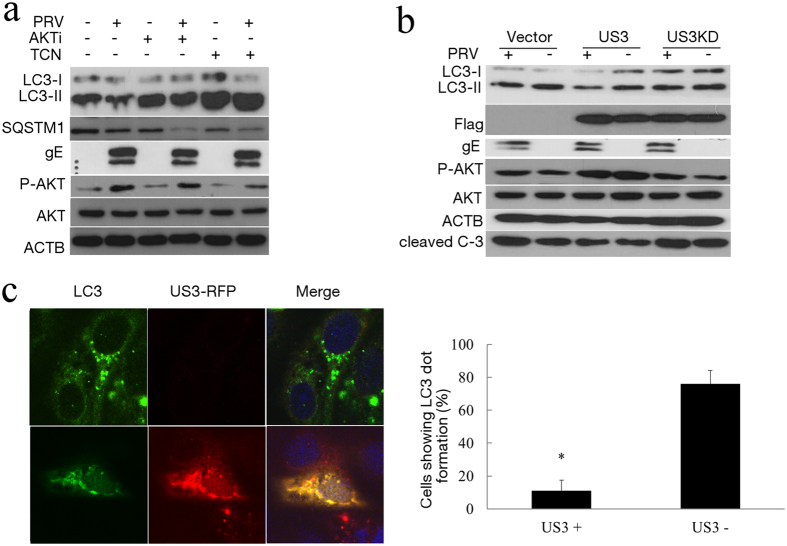
The US3 protein inhibits the autophagy response through activating PI3-K/AKT pathways. (**a**) To investigate the effects of drug treatment on levels of LC3-II and p-AKT in PRV-infected Vero cells, cells were pretreated with AKTi (10 μM) and triciribine (1 μM), then infected with PRV for 10 h. The cell extracts were analyzed by immunoblotting. (**b**) Vero cells were transfected with an empty vector, US3 or kinase-dead US3 for 24 h before PRV infection. At 10 hpi, the cells were analyzed by Western blotting. (**c**) Vero cells were transfected with plasmid RED-US3. After rapamycin treatment, the cells were fixed, and endogenous LC3 was analyzed with an immunoconfocal method as described before. Cells showing LC3 puncta were quantified. Twenty cells were analyzed per assay. The data represent the mean ± SD of three independent experiments. One-way ANOVA test; *P < 0.05. Gels were run under the same experimental conditions. For better clarity and concise presentation, cropped blots are shown. The raw uncropped images can be found in Supplementary Fig. S6.

US3 is a conserved, multifunctional alphaherpesvirus protein. It was reported that US3 protein kinase could activate PI3-K/AKT pathways to suppress apoptosis in PRV-infected cells^28^. Phosphorylated AKT (p-Ser473) participates in the PI3K/AKT/mTORC1 pathway to induce autophagy^40^,^41^. Therefore, we determined the phosphorylation of AKT in PRV-infected cells. Under basal conditions, AKT-S473 was moderately phosphorylated. After PRV infection, phospho-AKT-S473 increased compared to mock-infected cells (Fig. 1b,c). Because AKT is generally considered to be a negative regulator of autophagy, AKT inhibitors were used to test the involvement of AKT in autophagy induction during PRV infection. Our data showed that the phosphorylation of AKT was inhibited by AKTi and triciribine (TCN) in both PRV-infected and non-infected cells. AKTi increased the level of LC3-II in PRV-infected cells (lanes 1 and 3) and mock-infected cells (lanes 2 and 4). These results suggested that the AKT inhibitor could increase the autophagy level in PRV-infected cells. Triciribine had the same effect on autophagy induction (Fig. 8a). These results supported the hypothesis that PRV infection might inhibit autophagy through activation of the PI3K/AKT pathway.

We further analyzed whether US3 has an effect on autophagy during PRV infection. We expressed US3 protein and assessed the expression of LC3, P-AKT and US3 using immunoblotting analysis. We found that US3 expression increased the phosphorylated AKT and reduced the level of LC3-II in both mock infected (Fig. 8b, lanes 1 and 3) and PRV-infected cells (Fig. 8b, lanes 1 and 3). To test whether the kinase function of US3 is required to inhibit autophagy, a kinase-dead US3 variant was used, the result showed that kinase-dead US3 did not affect the activity of AKT nor inhibit autophagy. This result further confirmed that US3 inhibits autophagy through its kinase function to induce AKT phosphorylation. We also tested the anti-apoptotic activity of US3 by cleaved-caspase 3 analyses and apoptotic ELISA, and US3 decreased the activity of caspase and apoptosis as reported.

We then expressed US3 as a fusion protein with red fluorescent protein and analyzed the accumulation of endogenous LC3 dots in cells treated with rapamycin to introduce autophagy. LC3 puncta was visualized by the anti-LC3 antibody. As expected, the LC3 puncta was rarely detected in US3-RFP positive cells, as shown in Fig. 8c. Cells showing LC3 dot formation in both US3 positive and negative expression were quantified. As shown in Fig. 8c, autophagy was clearly inhibited in US3positive cells. These data indicated that US3 expression could inhibit autophagosome formation. Taken together, PRV tegument protein US3 inhibited the autophagy response by activating PI3-K/Akt pathways.

## Discussion

A growing number of viruses have been shown to affect autophagy^7^,^42^,^43^. In particular, there is a complex relationship between herpesviruses and autophagy. PRV is a large, complex virus that develops an interaction with host cells and establishes a life-long persistent infection. As an important pathogen in swine, the role of autophagy on the pathogenicity of PRV has not been elucidated. In the present study, we are the first to describe the interaction between PRV and autophagy. We showed that PRV infection resulted in the induction of autophagy without the requirement of viral replication during the early stages of infection. Subsequently, PRV replication reduced autophagy in several permissive cell lines before the detection of viral protein gE. Using shRNA to knockdown the ATG proteins in Vero cells, we demonstrated that PRV enhanced the infection with an increased viral titer and gene expression in autophagy-inhibited cells. We identified new functions of PRV tegument protein US3, which inhibits autophagy response by activating PI3-K/Akt pathways. These findings suggest that PRV is likely to have evolved strategies to antagonize host autophagy to facilitate its replication. Our studies extend the understanding of autophagy in herpesvirus.

We compared the change in autophagy progression after different PRV infection titers at MOIs of 0.1 and 10. When cells were infected with a titer at an MOI of 0.1 (titer specific to each cell line), the decreased autophagy level was detected until 12 hpi while the decreased autophagy level was detected at approximately 4 hpi when cells were infected with PRV at an MOI of 10. According to classic one-step growth experiments, an MOI of 10 was performed and new expression of the protein gE was detected at approximately 6 hpi. Cells were not actively infected at an MOI of 0.1, and viral replication was delayed with late growth kinetics. Figure 1 shows that the different effects on LC3 modification and SQSTM1/p62 degradation were consistent with the accumulation of PRV gE protein along with replication of the virus. Infection titers at MOIs of 0.1 and 10 had different results for the temporal progression of autophagy levels suggesting that autophagy was inhibited along with viral replication.

There is a large body of previous work investigating the role of autophagy in herpesvirus infections. Previous studies have shown a close and complex relationship between herpesviruses and the autophagy pathway. For example, PKR-dependent autophagic degradation of HSV-1 protects the cell^21^. However, HSV-1 encodes two proteins that inhibit autophagy, ICP34.5 and US11, to evade xenophagy^20^,^44^. Autophagy plays an important role in HSV-1 replication through ICP34.5, which contributes to virulence by preventing the xenophagic destruction of virions. Another important pathogen, VZV encodes the smallest genome among human herpesviruses, lacking homologs of both HSV-1 ICP34.5 and US11^45^. It was previously observed that VZV-induces autophagy associated with features that are common to ER stress and the unfolded protein response (UPR)^46^,^47^,^48^, and autophagy facilitates VZV glycoprotein biosynthesis and processing^48^. HCMV has been shown to affect autophagy; however, the exact nature of this relationship remains controversial^22^. Our studies suggest that PRV infection inhibits autophagy, which likely benefits viral replication. Interestingly, PRV lacks homologs of both HSV-1 ICP34.5 and US11.

We showed that the US3 protein is involved in the inhibition of the autophagy process for the first time in this study. It has been reported that US3 protein kinase suppresses apoptosis through the activation of the PI3-K/AKT and NF-ĸB pathways^28^. AKT, which is also known as Protein Kinase B (PKB), is a serine/threonine-specific protein kinase that plays a key role in multiple cellular processes, such as glucose metabolism, apoptosis, cell proliferation, transcription and cell migration^49^,^50^. Since phosphorylated AKT (p-Ser473) participates in the PI3K/AKT/mTORC1 pathway to induce autophagy^40^,^41^, we determined the phosphorylation of AKT that occurred in PRV-infected cells and participated in the inhibition of autophagy. The present study shows for the first time that the US3 protein has both anti-autophagy and anti-apoptosis functions, and these functions are kinase-dependent, which suggests that US3 plays an important role in the pathogenicity of PRV.

In our experiment, AKT inhibitors were used to confirm the autophagy activity of AKT. However, AKT is a multifunctional protein and plays role in virus entry^51^. There was a decrease in PRV replication in cells that were treated with AKT inhibitors. Thus, we tested the replication of PRV in ATG5 knockdown cells, and the results suggested that AKT inhibition decreased PRV replication mainly through its effect on autophagy. In conclusion, AKT inhibitors can be used to prove that the activity of AKT results in an inhibition of autophagy in PRV-infected cells. Our unpublished data also showed that the autophagy level was decreased to some degree, even in the absence of US3 protein. The results suggest that PRV had other means to manipulate the autophagy response.

In this study, we found that PRV could induce autophagy very soon after infection, even in the absence of any viral protein expression. A previous study on HSV-1 and HCMV showed that autophagy could be induced by the presence of foreign DNA within cells^23^. Another paper demonstrated that HSV-1 DNA was present in the cytosol following HSV-1 infection and induced autophagy in non-permissive cells in an IFN gene-dependent manner^30^. In bacteria, there was also a report that demonstrated *M. tuberculosis* extracellular DNA could induce autophagy by activating the host DNA-sensing pathway^52^. There are two hypotheses that either viral DNA or proteins on virions induced the autophagy response. Further investigation is required to identify the viral component(s) responsible for PRV-induced autophagy.

The herpesvirus viral genes can be subdivided into at least three classes of successively expressed transcripts, including immediate-early genes, early genes and late genes^1^,^21^,^53^. PRV has only one immediate early gene, IE180, which acts as the master switch of the PRV transcriptional cascade^54^. A reporter was used to demonstrate that the immediate-early protein IE180 of PRV is able to interfere with eIF2α phosphorylation, which plays an important role in the activation of autophagy^20^,^55^. Whether IE180 affects autophagy requires more detailed examination. Deleting PRV-encoded proteins that inhibit autophagy may shed light on the intracellular molecular mechanisms. However, IE180 is critical for the replication of PRV.

In conclusion, we have shown that PRV inhibits autophagy and that autophagy reduced PRV infection, suggesting a form of xenophagy. Further studies on the autophagy process will expand our understanding of PRV pathogenesis and provide insights for the development of novel antiviral strategies against PRV infection.

## Materials and Methods

### Cells and viruses

Vero, NIH-3T3 and PK-15 cells were cultured in Dulbecco’s modified Eagle medium (DMEM) (Life Technologies, 11995) supplemented with 10% fetal bovine serum (FBS) (Gibco-BRL Life 20 Technologies, 10099-141). The PRV strain HeN1 (1.2 × 10^7^ PFU/ml) was isolated and stored in our laboratory. The PRV stock was produced on a Vero cell monolayer and purified using sucrose density gradient centrifugation. PRV was UV-inactivated through UV irradiation of the virus inoculum in a dish on ice with 1,000 mJ/cm^2^ using the CL-1000 UV Cross-linker (UVP, Inc.) as previously described^55^.

### Chemicals, antibodies, and other reagents

Rapamycin (R0395), cycloheximide (CHX, A6185), AKT Inhibitor (A6730), triciribine (t3830), 3-MA (M9281), anti-β-actin antibody (A3853), and anti-LC3 antibody (L8918) were obtained from Sigma-Aldrich (Shanghai, China). Anti-AKT, anti-phospho-AKT, anti-ATG5 (6230), and anti-cleaved caspase 3 (Asp175) (9664) antibodies were obtained from Cell Signaling. The anti-gE antibody and anti-US3 antibody were produced from immunized mice. FITC-conjugated goat anti-mouse secondary antibodies and tetramethyl rhodamine isothiocyanate (TRITC)-conjugated goat anti-rabbit secondary antibodies were purchased from Zhongshan Jinqiao, China. The gene for US3 was amplified using the primers listed in Table S1 and cloned into the pCAGGS vector (Addgene, USA) and the pDsRed-Express-N1 vector (BD Biosciences Clontech, USA). For kinase-dead US3, we generated several point mutation mutants, including a lysine to glycine substitution at position 136 (K136G) and an aspartate to alanine substitution at position 223 (D223A), and a combination of these two positions. The primers are listed in Table S1. The nucleotide sequences of the plasmids encoding US3 and kinase-dead US3 were confirmed to ensure that the correct clones were used in the study. Plasmid GFP-LC3 was kindly provided by Professor William Jackson.

### Cell culture and virus infection

Cells were infected with PRV at an MOI of 0.1 or 10 as indicated or were mock infected with phosphate-buffered saline (PBS). After incubation for 1 h at 37 °C, the unbound virus was removed by washing the cells three times with PBS. The cells were then cultured in DMEM supplemented with 2% FBS at 37 °C for the indicated times. For the autophagy induction or inhibition experiments, cells were treated with different concentrations of rapamycin or 3-MA dissolved in dimethyl sulfoxide before/after virus infection.

### Transfection

The cells were seeded at a density of 1 × 10^6^ cells/ml on glass coverslips placed in 6-well tissue culture plates (Corning Glass Works, Corning). The following day, the cells were transfected at 80% confluence with the plasmid at 3.0 μg/well using the transfection reagent (Roche) according to the manufacturer’s instructions. The medium was replaced after 6 h with DMEM containing 2% FBS, and the cells were cultured for 24 h.

### RNA interference

Vero cells were grown to 80% confluence in six-well cell culture plates and then transiently transfected with the indicated small interfering RNA (shRNA) using transfection reagent (Roche) according to the manufacturer’s instructions. The silencing efficiency of the shRNA was detected by Western blotting analysis.

### Western blot analysis

Western blotting analysis was performed as previously described^56^. Briefly, the cells were washed with PBS three times and then scraped off the plate. The cells were then incubated on ice in cell lysis buffer (50 mM Tris-HCl, pH 7.4, 150 mM NaCl, 1% Triton X-100, 2 mM EDTA, 0.1% SDS, and 5 mM sodium orthovanadate) containing a protease inhibitor cocktail (Roche Molecular Biochemicals) and 0.1 mM PMSF for 2 h. The cell lysates were then centrifuged at 14,000× *g* for 20 min at 4 °C. The protein concentration was determined using the BCA assay (Thermo). Equal amounts of protein were separated on SDS-PAGE gels. The proteins in the gel were transferred to polyvinylidene fluoride (PVDF) membranes (ISEQ00010, Millipore, USA), which were then blocked with 5% nonfat dry milk in PBST for 2 h and incubated with different primary antibodies at 4 °C overnight. The next day, the membrane was incubated for 1 h with the appropriate secondary antibodies. Immuno-reactive bands were visualized using the enhanced chemiluminescence system (ECL, PerkinElmer Life Sciences).

### Confocal fluorescence microscopy

For confocal fluorescence, the cells were grown on coverslips and then infected with PRV as indicated at an MOI of 10. At 2 and 10 hpi, the cells were fixed with 4% paraformaldehyde for 30 min. The fixed cells were permeabilized with 0.1% Triton X-100 in PBS for 15 min and blocked with 3% bovine serum albumin in PBS for 2 h. Anti-LC3 antibodies were added to detect LC3 expression for 2 h, then incubated with FITC-conjugated goat anti-rabbit secondary antibodies. The second anti-gE antibodies were added to the fixed cells to detect gE expression for 2 h, and TRITC-conjugated goat anti-mouse secondary antibodies were added next. The nuclei were stained with 4′-6-Diamidino-2-Phenylindole (DAPI, D1306, Invitrogen, U.S.A.). To detect co-localization of LC3 and SQSTM1, anti-LC3 antibodies and anti-SQSTM1 antibodies and their respective secondary antibodies were used as indicated.

### Plaque formation assay

Ten-fold dilutions (from 10^2^ to 10^5^) of the viral culture supernatants were added to 6-well plates with a confluent monolayer of Vero cells. The plate was then incubated at 37 °C for 2 h with gentle agitation at every 30 min interval. The excess virus inocula were removed by rinsing the cells with PBS three times. Subsequently, overlay medium (2% low melting-point agarose with DMEM medium containing 2% FBS) was added to each well and further incubated at 37 °C with 5% CO _2_ for 3–4 days. The cells were then stained with 0.5% crystal violet, and the plaques were counted.

### *In vitro* entry studies

For entry kinetics, Vero cells were transfected with the indicated small interfering RNA (shRNA) for 24 h, and the cells were infected with virus (100 PFU per well) on ice for 1 h. After medium exchange, the cells were incubated for 0, 15, 30, or 60 min at 37 °C, and the extracellular virus was washed away with PBS. Subsequently, overlay medium (2% low melting-point agarose with DMEM medium containing 2% FBS) was added to each well and further incubated at 37 °C with 5% CO _2_. After 2 days, the cells were fixed, stained with crystal violet and the plaques were counted as described above in the plaque formation assay. The amount of virus that penetrated the cells was evaluated after the numbers of PFU were determined^38^,^39^,^57^.

### Cytotoxicity assay

Approximately 1 × 10^5^ Vero cells per well were seeded in a 96-well cell culture plate and cultured for 20 h at 37 °C under 5% CO_2_. The medium was replaced with fresh DMEM supplemented with 2% FBS with or without rapamycin or 3-MA, and the plates were incubated for an additional 24 or 48 h. Cytotoxicity was assayed by measuring the lactate dehydrogenase (LDH) release from cells with the CytoTox-One homogenous membrane integrity kit (G7890, Promega, USA) according to the manufacturer’s instructions. Vero cells were transfected with a shRNA vector for 48 h and a cytotoxicity assay was performed as described.

### DNA extraction and Q-PCR analysis

Quantitative real-time PCR (Q-PCR) was used to quantify PRV DNA. The primers that were used were complementary to the gB protein of PRV, which is an important PRV antigen that is abundantly expressed in infected cells. Cellular β-actin levels from the same DNA extract were used as an internal control. The viral copy number was expressed as a ratio of viral cDNA copies to cellular β-actin cDNA copies.

DNA from cultured cell samples was extracted as previously described^58^. Plasma DNA was extracted using a QIAamp DNA mini kit (Qiagen) according to the manufacturer’s instructions, and 1 μl of the DNA product was used as the template for the Q-PCR reaction with the primers listed in Table S1. The reaction was performed and analyzed following the manufacturer’s protocol (Takara).

### Statistical and image analysis

All statistical analyses were performed using a one-way ANOVA in SPSS version 16.0 software (SPSS Inc., Chicago, IL, USA). Data are expressed as the mean ± standard deviation (S.D.). A P value < 0.05 was considered statistically significant. The intensities of the Western blot bands were analyzed using Image J software (HIN).

## Additional Information

**How to cite this article**: Sun, M. *et al*. Pseudorabies virus infection inhibits autophagy in permissive cells *in vitro. Sci. Rep.* **7**, 39964; doi: 10.1038/srep39964 (2017).

**Publisher's note:** Springer Nature remains neutral with regard to jurisdictional claims in published maps and institutional affiliations.

## Supplementary Material

Supplementary Information
