# Supplementary material for: Pseudorabies virus infection inhibits autophagy in permissive cells in vitro
Source: Sci Rep. 2017 Jan 6;7:39964. doi: 10.1038/srep39964 (PMC5216355; doi:10.1038/srep39964)
Supplement: Supplementary Information [file srep39964-s1.pdf]

Pseudorabies virus infection inhibits autophagy in permissive cells *in vitro*

Mingxia Sun[a], Linlin Hou[a,b], Yandong Tang[a], Yonggang Liu[a], Shujie Wang[a], Jingfei Wang[a], Nan Shen[a], Tongqing An[a], Zhijun Tian[a], Xuehui Cai[a]*

Figure S1 PRV infection modified LC3 lipidation after infection

Figure S1 showed the raw uncropped images of figure1a,b and c.

Figure1a

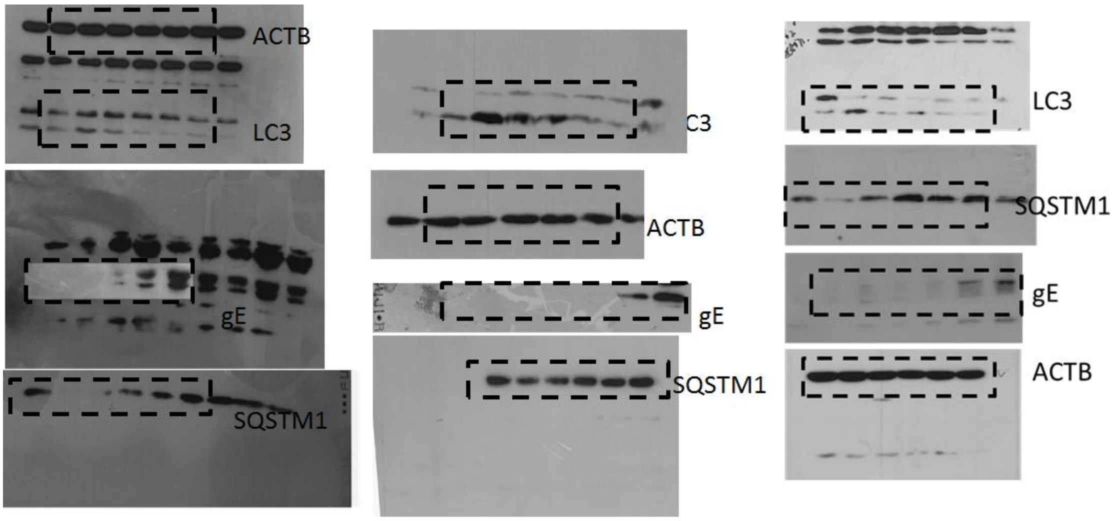

Figure1b

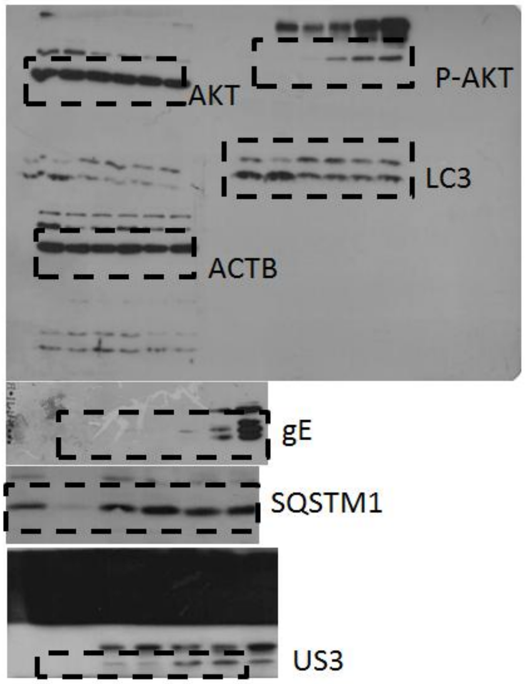

Figure1c

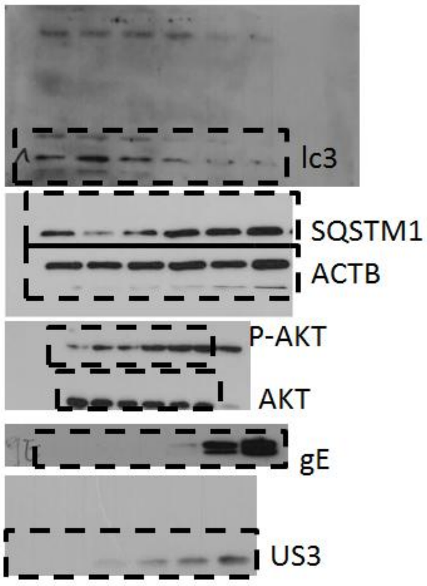

Figure S2 autopahgy flux in PRV infected cells

Figure S2 showed the raw uncropped images of figure4a.

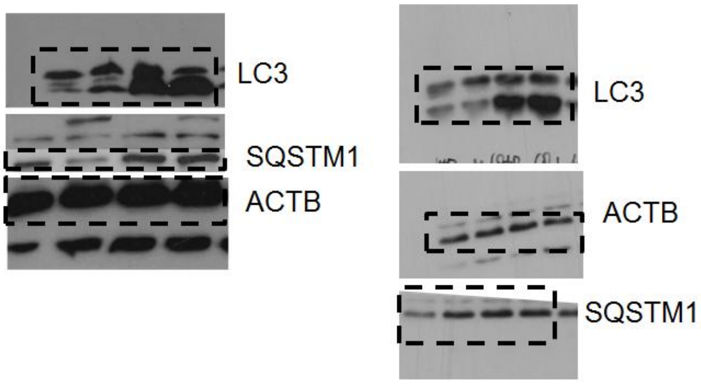

Figure S3 autophagy and viral replication

Figure S3 showed the raw uncropped images of figure5a and b.

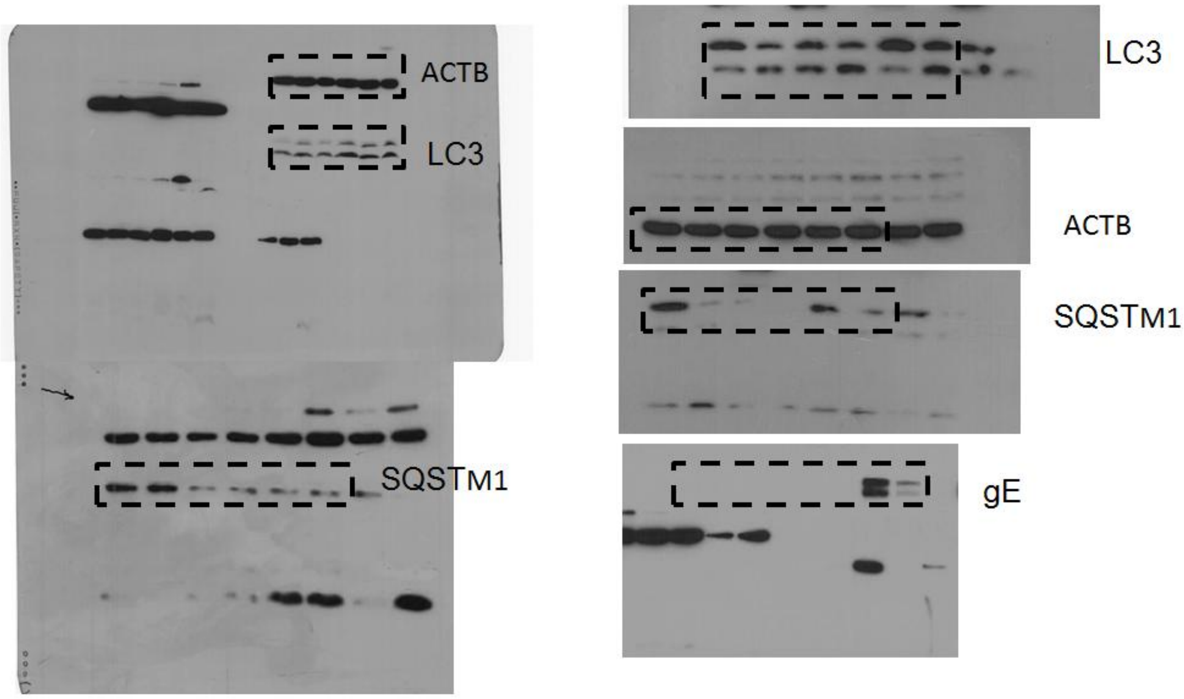

Figure S4 the effect of rapamycin and 3-MA on PRV infected cells

Figure S4 showed the raw uncropped images of figure6a and e.

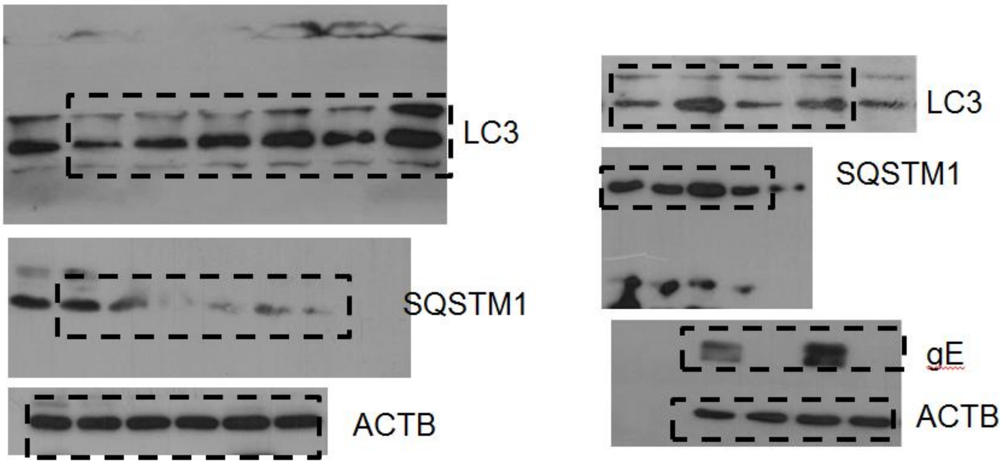

Figure S5 autophagy level after ATG genes silencing

Figure S5 showed the raw uncropped images of figure7a.

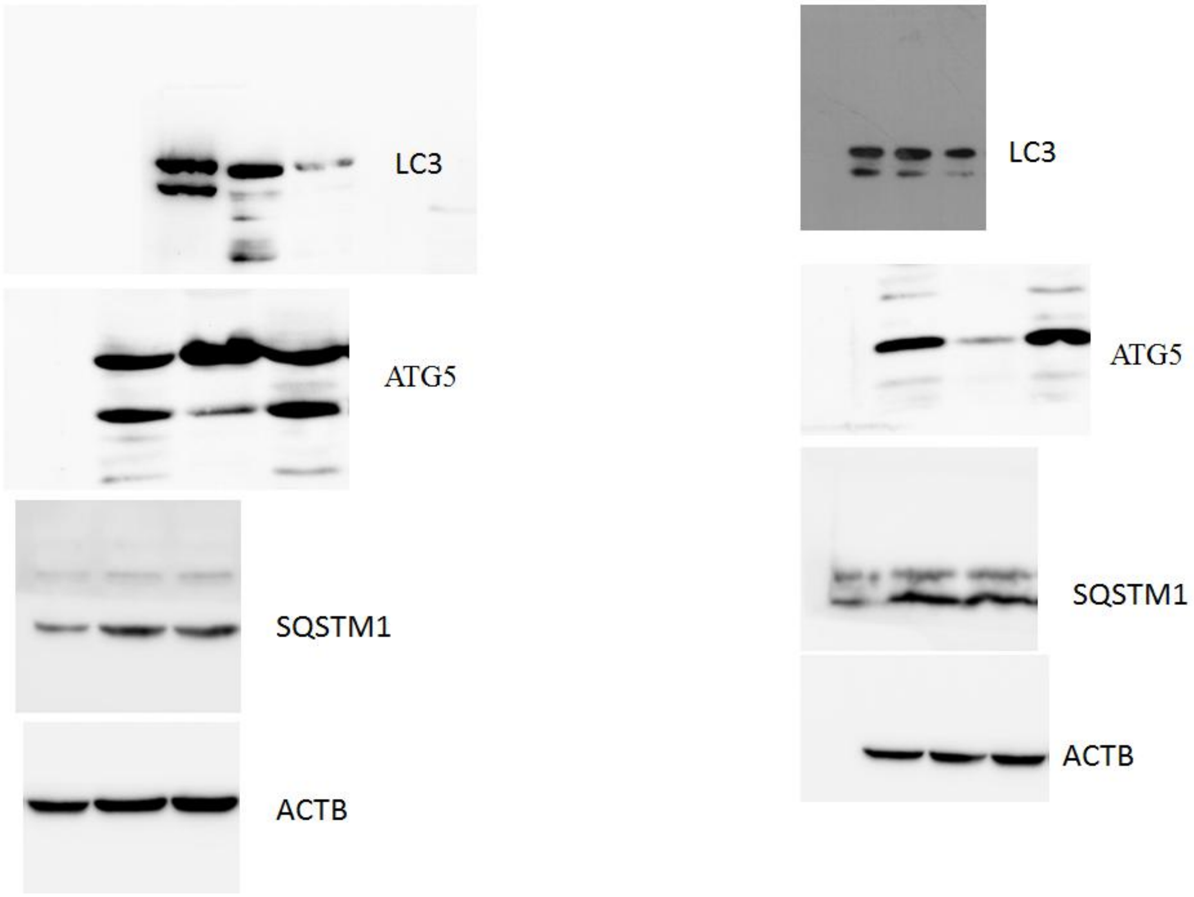

Figure S6 the US3 protein inhibits the autophagy response

Figure S2 showed the raw uncropped images of figure8a and b.

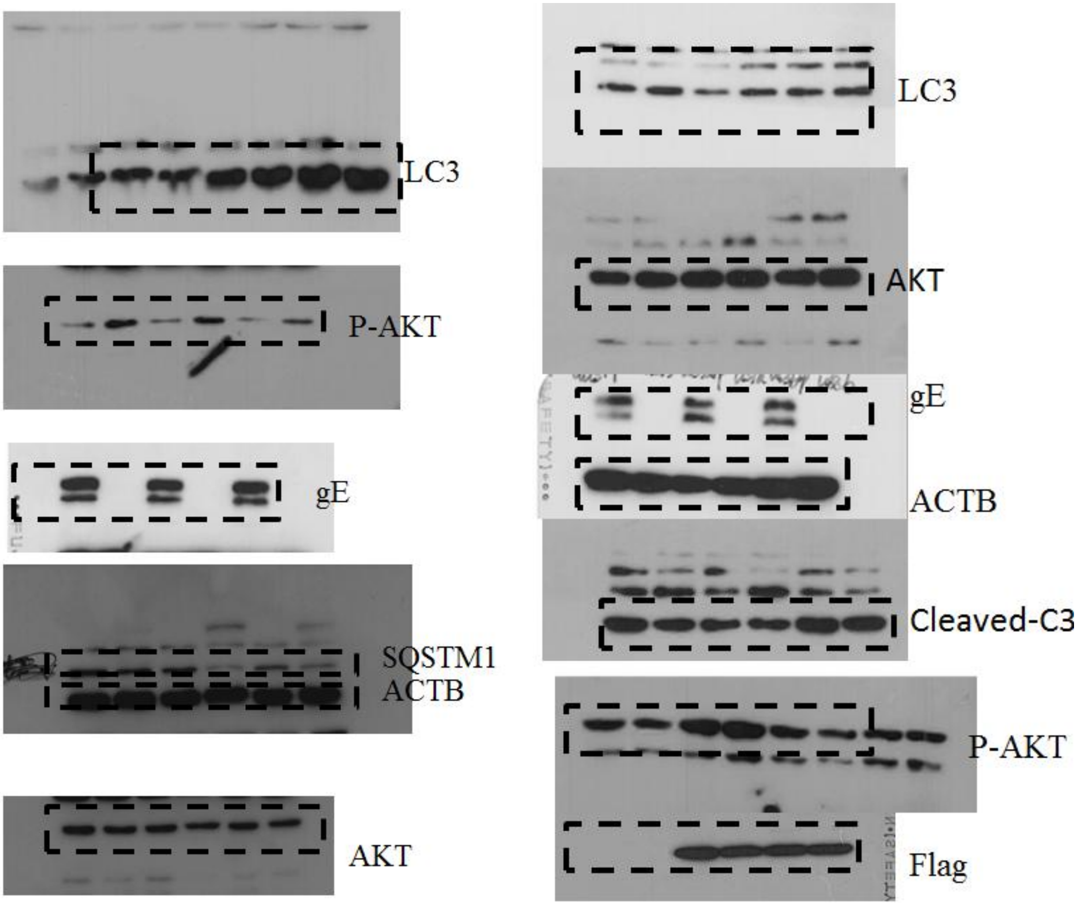

Figure S7 UV-inactivated PRV

The infectivity of inactivated PRV was detected by titration and IFA assay. The virus samples with 10-fold dilutions were added into 6-well plate with monolayer of Vero cells to test plaque formation. As shown in Figure S7, UV-inactivated PRV did not format plaque and there was no IFA signal specific for the gE protein of PRV, indicating that the inactivated PRV virion is not infective.

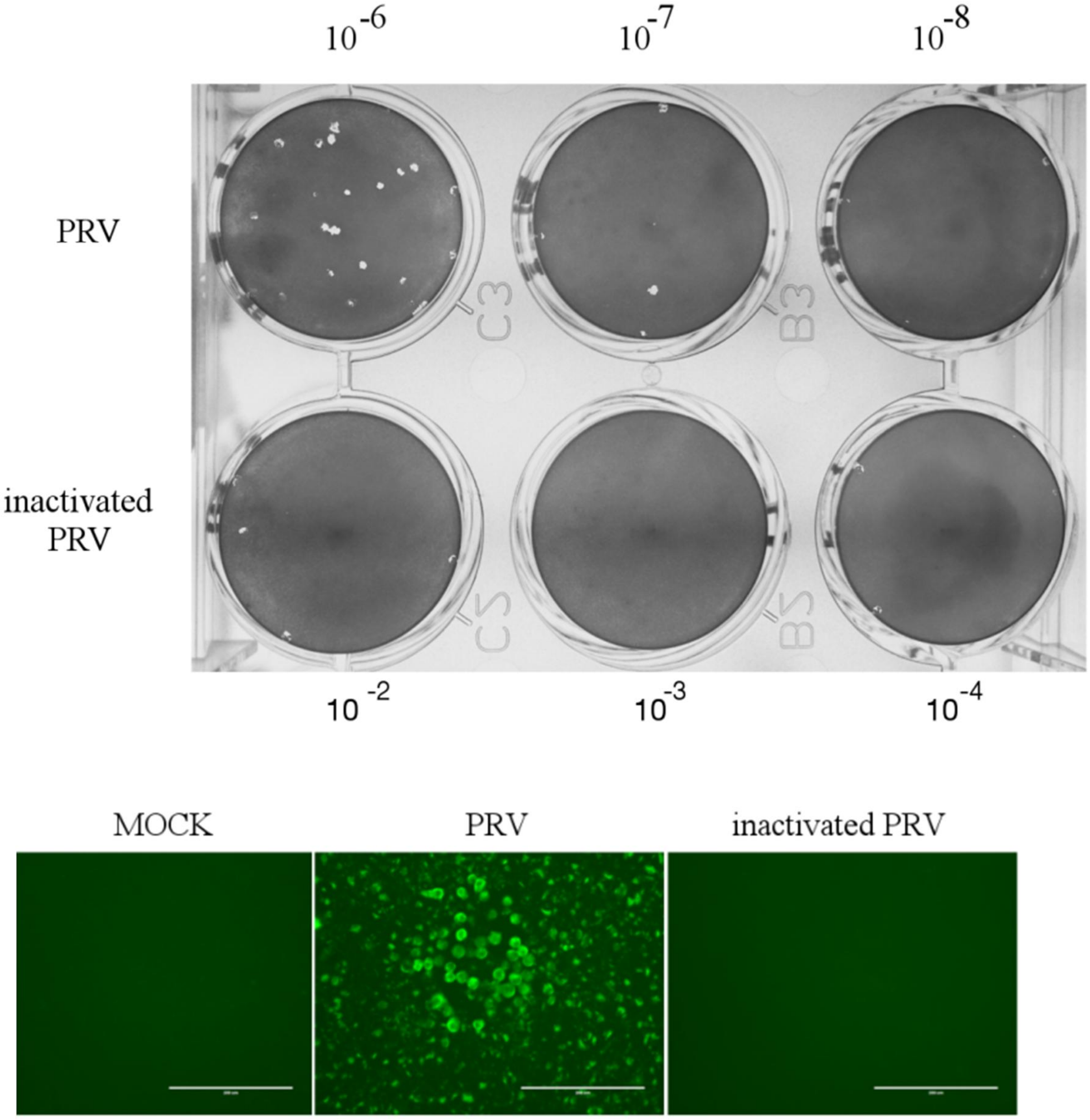

Supplementary Table S1

Table S1. The primer sequences used for Q-PCR and plasmids construction

Gene	Primer sequence (5'-3')
gB	Forward GTCACCTTGTGGTTGTTG
	Reverse CCACATCTACTACAAGAACG
β-actin	Forward CTTCCTGGGCATGGAGTCC
	Reverse GGCGCGATGATCTTGATCTTC
US3-FLAG	Forward ATTTAAGCTTGCCACCATGGCCGACGCCGGAATCCCCGACGAGA
	Reverse GGTTGAATTCTTACTTATCGTCGTCATCCTTGTAATCTACGGTCCACATTCC
US3-RFP	Forward CTAGCTAGCGCCACCATGGCCGACGCCGGAATCCCCGA
	Reverse TCCGGAATTCGTTATACGGTCCACATTCCAAAGTTG
US3(D223A)	Forward TACGACACCAAGGTCGCGGTCTGGGGTGCG
	Reverse CGCACCCCAGACCGCGACCTTGGTGTCGTA
US3(K136G)	Forward ACGGTGGTGCTGCAGGTGGGCCAGA
	Reverse TCTGGCCCACCTGCAGCACCACCGT
